# Microporous metal–organic framework with dual functionalities for highly efficient removal of acetylene from ethylene/acetylene mixtures

**DOI:** 10.1038/ncomms8328

**Published:** 2015-06-04

**Authors:** Tong-Liang Hu, Hailong Wang, Bin Li, Rajamani Krishna, Hui Wu, Wei Zhou, Yunfeng Zhao, Yu Han, Xue Wang, Weidong Zhu, Zizhu Yao, Shengchang Xiang, Banglin Chen

**Affiliations:** 1 Department of Chemistry, TKL of Metal- and Molecule-Based Material Chemistry, Collaborative Innovation Center of Chemical Science and Engineering (Tianjin), Nankai University, Tianjin 300071, China; 2 Department of Chemistry, University of Texas at San Antonio, One UTSA Circle, San Antonio, Texas 78249-0698, USA; 3 Van ‘t Hoff Institute for Molecular Sciences, University of Amsterdam, Science Park 904, Amsterdam 1098 XH, The Netherlands; 4 NIST Center for Neutron Research, Gaithersburg, Maryland 20899-6102, USA; 5 Advanced Membranes and Porous Materials Center, Physical Sciences and Engineering Division, King Abdullah University of Science and Technology, Thuwal 23955-6900, Saudi Arabia; 6 Key Laboratory of the Ministry of Education for Advanced Catalysis Materials, Institute of Physical Chemistry, Zhejiang Normal University, Jinhua 321004, China; 7 College of Chemistry and Chemical Engineering, Fujian Provincial Key Laboratory of Polymer Materials, Fujian Normal University, Fuzhou 350007, China

## Abstract

The removal of acetylene from ethylene/acetylene mixtures containing 1% acetylene is a technologically very important, but highly challenging task. Current removal approaches include the partial hydrogenation over a noble metal catalyst and the solvent extraction of cracked olefins, both of which are cost and energy consumptive. Here we report a microporous metal–organic framework in which the suitable pore/cage spaces preferentially take up much more acetylene than ethylene while the functional amine groups on the pore/cage surfaces further enforce their interactions with acetylene molecules, leading to its superior performance for this separation. The single X-ray diffraction studies, temperature dependent gas sorption isotherms, simulated and experimental column breakthrough curves and molecular simulation studies collaboratively support the claim, underlying the potential of this material for the industrial usage of the removal of acetylene from ethylene/acetylene mixtures containing 1% acetylene at room temperature through the cost- and energy-efficient adsorption separation process.

## Abstract (web summary)

The removal of acetylene from ethylene/acetylene mixtures is an industrially important but challenging task. Here the authors report a microporous metal–organic framework with optimized pore/cage space design and chemical functionalization, capable of removing acetylene from low concentration mixtures.

## Graphical abstract

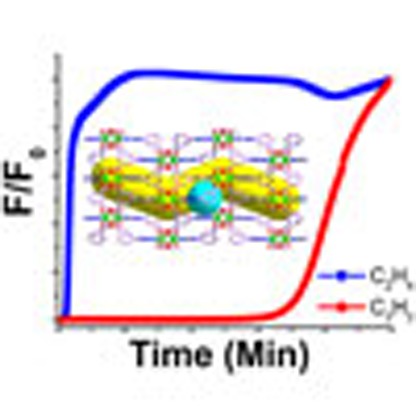

Ethylene is one of the most essential raw chemicals and widely used to produce polymers and other useful chemicals^1^. During the production of ethylene through the cracking of ethane, propane and heavier hydrocarbons, a small amount of acetylene as an impurity of about 1% is also generated. It is imperative that acetylene in the ethylene feed should be reduced to an acceptable level because acetylene has a deleterious effect on end products of ethylene: acetylene can cause a catalyst poison during ethylene polymerization and thus significantly affect the quality of the resulting polyethylene. Furthermore, acetylene can form solid metal acetylides, which can block the fluid stream and lead to explosion^2^.

Extensive efforts have been pursued to remove acetylene from ethylene/acetylene mixtures^3^,^4^. In the petrochemical industry, current commercial approaches include partial hydrogenation of acetylene into ethylene over a noble metal catalyst such as a supported Pd catalyst and solvent extraction of cracked olefins using an organic solvent such as DMF and acetone. Both of which have some drawbacks: the former process suffers from the need of noble metal catalyst and the loss of olefins due to the over hydrogenation to paraffins, while the latter wastes a significant amount of solvents. Porous materials through selective adsorption separation of acetylene over ethylene might provide an alternative cost- and energy-efficient approach for this industrially very important while quite challenging task, though this has not been fully explored.

Among diverse porous materials, the emerging microporous metal–organic frameworks (MOFs) are of particular interest and are important for gas separation and thus removal of acetylene from ethylene/acetylene mixtures. This is because the pores within microporous MOFs can be straightforwardly and rationally tuned to enforce their size selective sieving effects while their pore surfaces can be readily functionalized to induce preferential interactions with specific gas molecules^5^,^6^,^7^,^8^,^9^,^10^,^11^,^12^,^13^,^14^,^15^,^16^,^17^,^18^,^19^,^20^,^21^,^22^,^23^,^24^,^25^,^26^,^27^,^28^,^29^,^30^,^31^,^32^,^33^,^34^,^35^. Although such a potential has been speculated, MOFs for the separation of C_2_H_2_/C_2_H_4_ have not been fully explored. We realized the first microporous MOF for this challenging separation in 2011^36^. We have been further able to tune the micropores through the interplay of metalloligands and organic linkers, and thus to optimize separation selectivities. Although the separation selectivities of these MOFs for the separation of C_2_H_2_/C_2_H_4_ are quite high because of their extraordinarily high sieving effects; their extremely narrow pores have also limited their acetylene uptake, which eventually affects their overall performance for separation of C_2_H_2_/C_2_H_4_, as clearly demonstrated in the simulated breakthrough curves^37^. Further development led to the discovery of MOF-74 series for C_2_H_2_/C_2_H_4_ separation in 2012^38^,^39^. This series of MOFs has high densities of open metal sites, which can significantly enforce their high acetylene uptake^40^,^41^, but their pores are too large to introduce size-sieving effects. Furthermore, the open metal sites have quite strong interactions with ethylene molecules, so MOF-74 series have systematically quite low selectivities for C_2_H_2_/C_2_H_4_ separation. The ideal MOFs for C_2_H_2_/C_2_H_4_ separation are those with high C_2_H_2_/C_2_H_4_ sieving effects but without sacrificing acetylene uptake. From the MOF structural point of view, these MOFs should still have comparatively narrow pores to enforce their high sieving effects, but these narrow pores should still take up moderate amount of acetylene molecules. Furthermore, some additional intercrossed cage spaces will be required to exclusively bind acetylene molecules and thus to maximize acetylene uptake. It should be theoretically feasible for us to design and realize MOFs to meet these criteria; however, in the reality, it is still a daunting challenge. Recently, there has been some progress on microporous MOFs for C_2_H_2_/C_2_H_4_ separation; however, their pore structures still cannot meet the above mentioned criteria and their separation performances are comparable to established ones^42^,^43^. Here we report a microporous MOF [Cu(ATBDC)]·G (UTSA-100; H_2_ATBDC=5-(5-Amino-1*H*-tetrazol-1-yl)-1,3-benzenedicarboxylic acid; G=guest molecules), based on our extensive research endeavours on microporous MOFs for C_2_H_2_/C_2_H_4_ separation, which can indeed meet those mentioned criteria. UTSA-100 is thus superior to other MOFs, exhibiting highly efficient removal of acetylene from ethylene/acetylene mixtures containing 1% acetylene.

## Results

### Preparation and characterization of UTSA-100

The amino derivative of tetrazol-1,3-benzenedicarboxylic acid (5-(5-Amino-1*H*-tetrazol-1-yl)-1,3-benzenedicarboxylic acid, H_2_ATBDC) was prepared based on alkaline decomposition of the tetrazole ring and heterocyclization of the resulting *N*-arylcyanamides on interaction with ammonium azide generated *in situ*. Reaction of CuCl_2_·2H_2_O with H_2_ATBDC in the solvothermal condition at 353 K formed UTSA-100 as green block single crystals. It was formulated as [Cu(ATBDC)]·G (UTSA-100) by single-crystal X-ray diffraction (SXRD) studies, and the phase purity of the bulk material was independently confirmed by powder X-ray diffraction (PXRD) (Supplementary Figs 4 and 5). The desolvated [Cu(ATBDC)] (UTSA-100a) for the adsorption studies was prepared from the acetone-exchanged samples followed by the activation under ultrahigh vacuum at room temperature (296 K) for one day, and then at 358 K for another 3 days. The PXRD profile of desolvated UTSA-100a indicates that it maintains the crystalline framework structure (Supplementary Fig. 5).

**Figure 1:**
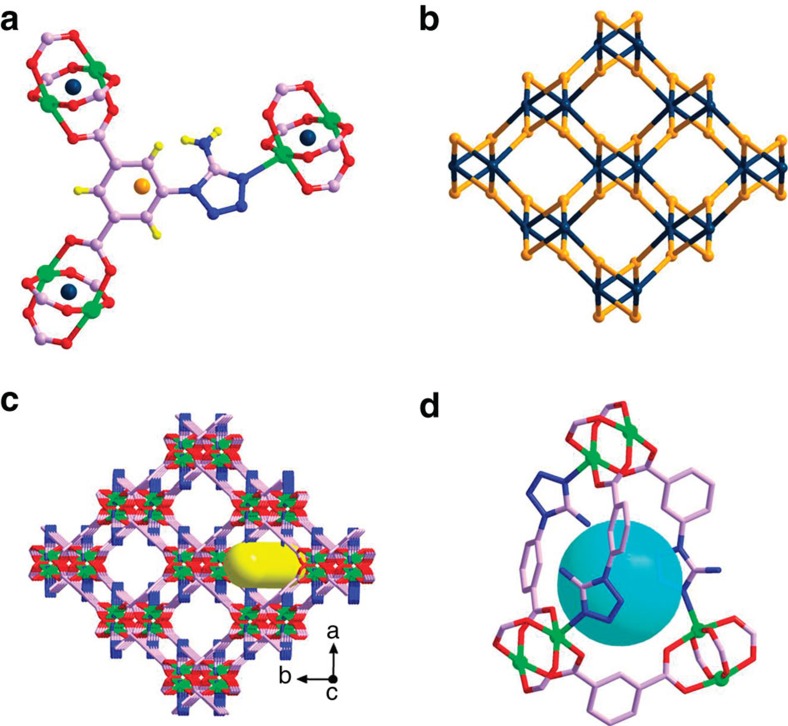
X-ray crystal structure of UTSA-100. (**a**) The coordination environment of organic ligand ATBDC^2−^ and Cu(II), and dinuclear copper(II) unit as a 6-connected node (purple balls) and ATBDC^2−^ as a 3-connected node (orange ball). (**b**) The framework topology of ***apo***-type (3,6)-connected network with Schläfli symbol {4.6^2^}_2_{4^2^.6^9^.8^4^}. (**c**) The 3D structure viewed along the *c* axis showing the 1D rhombic channels of about 4.3 Å in diameter. (**d**) The cage with a diameter of about 4.0 Å between 1D channels with window openings of 3.3 Å. Solvent molecules were omitted for clarity. Colour scheme: Cu, bright green; O, red; N, blue; C, orchid; and H, yellow.

X-ray single-crystal structure reveals that UTSA-100 has a three-dimensional framework with rhombic open zigzag nanochannels with amino and tetrazole functionalized wall running in the *c*-direction (Fig. 1; Supplementary Table 1; Supplementary Data 1). There are 6-connected binuclear Cu_2_(COO)_4_ units, which are bridged by 3-connected ATBDC^2−^ anions to form a (3,6)-connected ***apo***-type network with Schläfli symbol {4.6^2^}_2_{4^2^.6^9^.8^4^} (Fig. 1b). The one-dimensional (1D) open zigzag channels with a diameter of about 4.3 Å are filled with the disordered solvent molecules (DMF and CH_3_OH), and there are small cages with the diameter of about 4.0 Å between the 1D channels with window openings of 3.3 Å (Fig. 1d). The calculated solvent accessible volume of UTSA-100a is 51.0%, estimated using the PLATON program^44^.

### Microporous nature of UTSA-100

To assess the permanent porosity, the acetone-exchanged UTSA-100 was further activated under high vacuum to obtain the desolvated UTSA-100a. The porosity of UTSA-100a was evaluated by N_2_-gas sorption at 77 K. The type I isotherm shows a very sharp uptake at *P*/*P*_*0*_<0.1, which clearly indicates its microporous nature (Supplementary Fig. 7a). The nitrogen physisorption of UTSA-100a reaches a plateau at around *P*/*P*_0_=0.1, the saturation uptake is 257.6 cm^3^ g^−1^ and the corresponding specific pore volume is 0.399 cm^3^ g^−1^. The Langmuir (Brunauer, Emmett and Teller ) surface area based on the N_2_ adsorption isotherm at 77 K is 1,098 (970) m^2^ g^–1^ for UTSA-100a, within the pressure range of 0.05<*P*/*P*_0_<0.3 (Supplementary Fig. 7b–d). The experimental determined Brunauer, Emmett and Teller surface of 970 m^2^ g^–1^ matches well with the simulated one (913 m^2^ g^–1^) from its crystal structure data.

### Sorption of acetylene and ethylene within UTSA-100a

**Figure 2:**
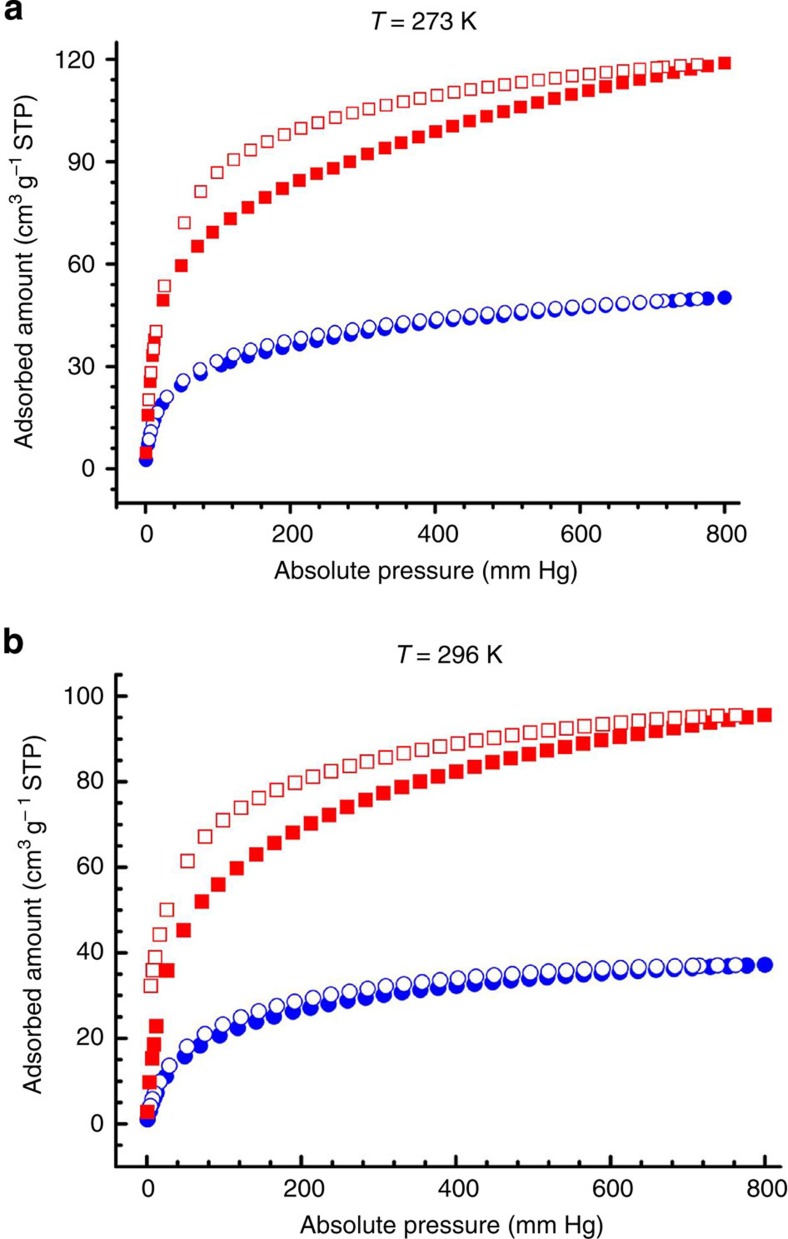
Gas sorption isotherms on the activated UTSA-100a. (**a**) Acetylene (red) and ethylene (blue) sorption at 273 K. (**b**) Acetylene (red) and ethylene (blue) sorption at 296 K. Adsorption and desorption branches are shown with closed and open symbols, respectively.

The unique pore structure encouraged us to examine the capacities of UTSA-100a for the selective separation of C_2_H_2_/C_2_H_4_. The low-pressure sorption isotherms of acetylene and ethylene were collected at 273 and 296 K, respectively. At 296 K and 1 atm, the acetylene and ethylene uptake amounts of UTSA-100a are 95.6 and 37.2 cm^3^ g^−1^, respectively (Fig. 2). This is really encouraging: the acetylene uptake of UTSA-100a is moderately high, while ethylene uptake is much lower. As shown in Table 1, the C_2_H_2_/C_2_H_4_ uptake ratio of 2.57 in UTSA-100a is systematically higher than the examined MOFs except M'MOF-3a with very narrow pores, indicating its bright promise for the C_2_H_2_/C_2_H_4_ separation.

The measured pure component isotherm data for acetylene and ethylene on UTSA-100a were fitted with the dual-Langmuir–Freundlich isotherm model. The fitted parameter values are presented in Supplementary Table 2. As illustration of the goodness of the fits, Supplementary Fig. 8 presents a comparison of component loadings for acetylene and ethylene at 296 K in UTSA-100a with the isotherm fits. The fits are excellent for both components over the entire pressure range.

The binding energy of acetylene is reflected in the isosteric heat of adsorption, *Q*_st_, defined as


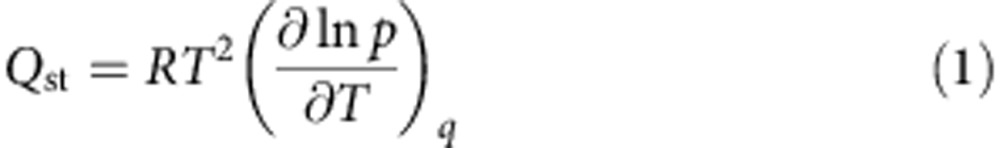


Supplementary Figure 10 presents a comparison of the heats of adsorption of acetylene in UTSA-100a with five other representative MOFs (M'MOF-3a, MgMOF-74, CoMOF-74, FeMOF-74 and NOTT-300); the calculations are based on the use of the Clausius–Clapeyron equation. We note that values of *Q*_st_ in UTSA-100a and M'MOF-3a are significantly lower than that of MOFs with coordinately unsaturated metal atoms FeMOF-74, CoMOF-74, and MgMOF-74. The value of *Q*_st_ in UTSA-100a is also lower than for NOTT-300. This implies that the regeneration energy requirement of UTSA-100a will be lower than that of FeMOF-74, CoMOF-74, MgMOF-74 and NOTT-300.

### IAST calculations of adsorption selectivities

**Figure 3:**
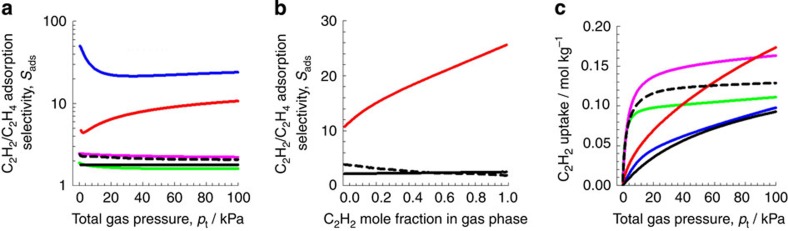
IAST calculations for binary C_2_H_2_/C_2_H_4_ mixture on the MOFs. (**a**) C_2_H_2_/C_2_H_4_ adsorption selectivity, and (**c**) uptake capacity of C_2_H_2_ for adsorption from C_2_H_2_/C_2_H_4_ mixture containing 1% C_2_H_2_. The total bulk gas phase is at 296 K and 100 kPa. The partial pressures of C_2_H_2_, and C_2_H_4_ are, respectively, *p*1=1 kPa, *p*2=99 kPa. (**b**) IAST calculations of the C_2_H_2_/C_2_H_4_ adsorption selectivity for FeMOF-74, NOTT-300, and UTSA-100a as a function of the mole fraction of C_2_H_2_ in the gas phase. The total gas pressure is constant at 100 kPa. The data for FeMOF-74 is at the temperature of 318 K; this is the lowest temperature used in the isotherm measurements of Bloch *et al*.^38^. The data for NOTT-300 is at 293 K. M'MOF-3a (blue), MgMOF-74 (pink), CoMOF-74 (green), FeMOF-74 (black dash), NOTT-300 (black) and UTSA-100a (red).

Analysis of the pure component isotherms at 296 K via ideal adsorbed solution theory (IAST)^45^ was carried out to estimate the selectivity between acetylene and ethylene. We consider the separation of binary ethylene/acetylene mixtures containing 1%, that is, 10,000 p.p.m., of acetylene, in the mixture; this composition is typical of industrial mixtures. Figure 3a presents the IAST calculations of the ethylene/acetylene adsorption selectivity, defined by


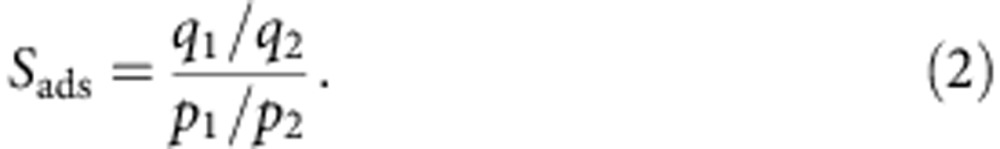


The highest adsorption selectivity is with M'MOF-3a, and this is followed by UTSA-100a. MOFs with coordinately unsaturated metal atoms MgMOF-74, CoMOF-74 and FeMOF-74 have selectivities that are in the range of 1.6 to 2.2. NOTT-300 has selectivities in the range 1.8–2.1.

In the paper by Yang *et al*.^42^, the ethylene/acetylene mixtures for NOTT-300 are compared with FeMOF-74 for varying mole fraction of acetylene in the gas phase, keeping the total gas phase pressure constant at 100 kPa. It must be remarked that in industrial practice, the compositions of acetylene in the gas phase are <1%. Also, acetylene is selectively adsorbed from ethylene/acetylene mixtures, and the bulk gas phase compositions will vary from 1% at the inlet to the desired 40 p.p.m. limit at the outlet of the fixed-bed absorber. Nevertheless, in order to compare our IAST calculation methodology with those of Yang *et al*., we carried out similar comparison, also including UTSA-100a; the results are shown in Fig. 3b. Our IAST selectivity calculations for NOTT-300 and FeMOF-74 agree reasonably well with those of Yang *et al.,* We note that UTSA-100a has significantly higher selectivity than both NOTT-300 and FeMOF-74.

As shown in Fig. 3c, the gravimetric uptake capacity of acetylene in UTSA-100a for adsorption from C_2_H_2_/C_2_H_4_ mixtures containing 1% C_2_H_2_ is compared with other five MOFs (M'MOF-3a, MgMOF-74, CoMOF-74, FeMOF-74 and NOTT-300). At a total gas phase pressure of 100 kPa, the hierarchy of uptake capacities for acetylene is UTSA-100a>MgMOF-74>FeMOF-74>CoMOF-74>M'MOF-3a≈NOTT-300.

### Ethylene/acetylene breakthrough simulations and experiments

**Figure 4:**
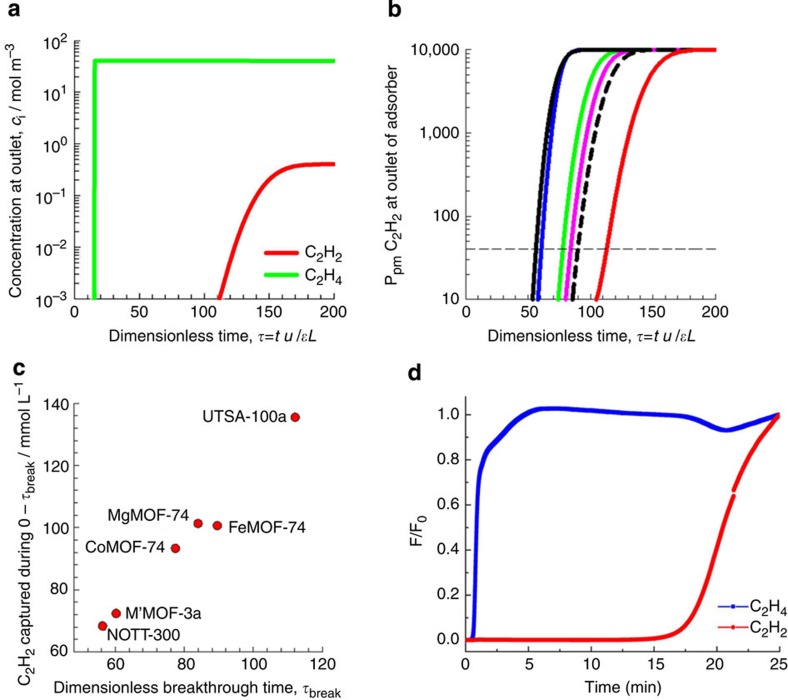
Simulative and experimental column breakthrough experiments. (**a**) Transient breakthrough curve of C_2_H_2_/C_2_H_4_ mixture containing 1% C_2_H_2_ in an adsorber bed packed with UTSA-100a. The partial pressures of C_2_H_2_, and C_2_H_4_ in the inlet feed gas mixture are, respectively, *p*_1_=1 kPa, *p*_2_=99 kPa. For the breakthrough simulations, the following parameter values were used, *L*=0.12 m; *ɛ*=0.75; *u*=0.00225, m s^−1^. (**b**) Ppm C_2_H_2_ in the outlet gas of an adsorber bed packed with UTSA-100a and various MOFs. M'MOF-3a (blue), MgMOF-74 (pink), CoMOF-74 (green), FeMOF-74 (black dash), NOTT-300 (black), and UTSA-100a (red). (**c**) Plot of C_2_H_2_ captured per L of adsorbent (< 40 ppm C_2_H_2_ in outlet gas), during the time interval 0–*τ*_break_, plotted as a function of the time interval *τ*_break_. (**d**) Experimental column breakthrough curve for C_2_H_2_/C_2_H_4_ mixed gas containing 1% C_2_H_2_ over UTSA-100a. The experiment temperatures are 296 K except FeMOF-74 (318 K) and NOTT-300 (293 K).

We carried out breakthrough simulations for C_2_H_2_/C_2_H_4_ (1:99, v/v) mixture, whose composition is typical of industrial mixtures, in a fixed-bed to demonstrate the feasibility of purification of ethylene in a Pressure Swing Adsorption operation. The transient breakthrough simulations show the concentrations of C_2_H_2_/C_2_H_4_ exiting the adsorber packed with UTSA-100a as a function of the dimensionless time, *τ* (Fig. 4a). Analogous breakthrough simulations were performed for M'MOF-3a, MgMOF-74, CoMOF-74, FeMOF-74 and NOTT-300. On the basis of the gas phase concentrations, we can calculate the impurity level of acetylene in the gas mixture exiting the fixed-bed packed with six different MOFs. Figure 4b shows the p.p.m. C_2_H_2_ in the outlet gas mixture exiting an adsorber packed with M'MOF-3a, MgMOF-74, CoMOF-74, FeMOF-74, NOTT-300 and UTSA-100a. At a certain time, *τ*_break_, the impurity level will exceed the desired purity level of 40 p.p.m. (indicated by the dashed line), that corresponds to the purity requirement of the feed to the polymerization reactor. The adsorption cycle needs to be terminated at that time *τ*_break_ and the regeneration process needs to be initiated. From a material balance on the adsorber, the amount of acetylene captured during the time interval 0–*τ*_break_ can be determined. The amount of acetylene captured in UTSA-100a during the time 0–*τ*_break_ is 137.6 mmol l^−1^, which is the best one among the compared six MOFs and approximately twice that of NOTT-300 (Supplementary Table 5). A plot of the amount of acetylene captured plotted as a function of the time interval *τ*_break_ is presented in Fig. 4c. The hierarchy of acetylene capture capacities is UTSA-100a>MgMOF-74>FeMOF-74>CoMOF-74>M'MOF-3a>NOTT-300. The significantly superior performance of UTSA-100a is attributable to a combination of high adsorption selectivity and high uptake capacity. M'MOF-3a has the highest selectivity but the lowest uptake capacity; this results in poorer performance in the industrial fixed-bed adsorber. It needs to be pointed out that the separations in fixed-bed adsorbers are determined by a combination of adsorption selectivity and uptake capacity. Transient breakthrough simulations can provide us some guidance on the potentials of materials for their real industrial applications. They can also offer more information than IAST calculations, and help us to make a clear comparison with other MOFs.

To evaluate the performance of UTSA-100a in the actual adsorption-based separation and purification processes, breakthrough experiments were performed in which an C_2_H_2_/C_2_H_4_ (1:99, v/v) mixture was flowed over a packed bed of UTSA-100a solid with a total flow of 2 ml min^−1^ at 296 K. As shown in Fig. 4d, the separation of C_2_H_2_/C_2_H_4_ (1:99, v/v) mixture through the column packed bed of UTSA-100a solid can be efficiently achieved. To the best of our knowledge, this is the first example of porous materials whose separation for C_2_H_2_/C_2_H_4_ (1:99, v/v) has been clearly established by experimental breakthrough, enabling UTSA-100a to be a potential high-performance material for real industrial ethylene purification application.

### Pore structure analysis and first-principles calculations

**Figure 5:**
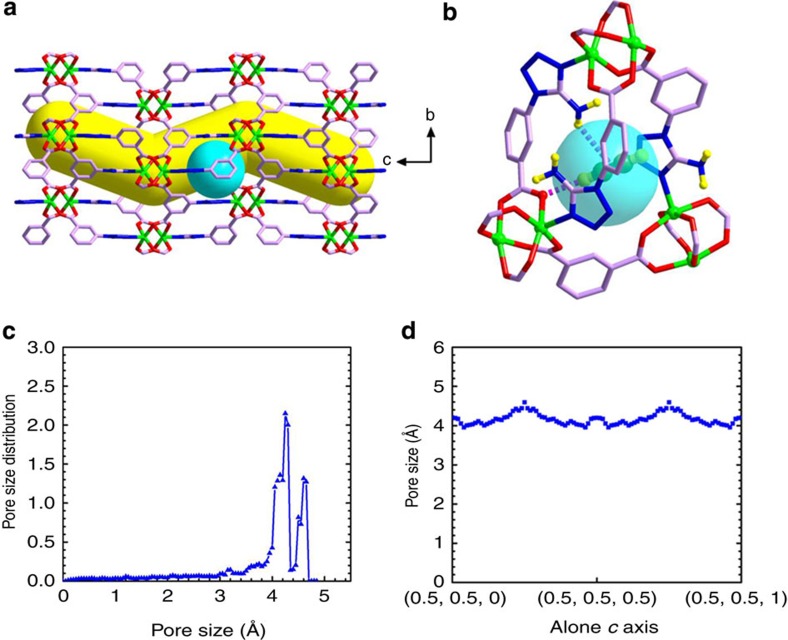
The pore structure of UTSA-100 and the C_2_H_2_ binding site. (**a**) The pore structure showing the zigzag channels along the *c* axis and the cage with a diameter of about 4.0 Å in the pore wall with window openings of 3.3 Å. (**b**) The acetylene sits right at the small cage connecting two adjacent channel pores. (multiple-point interactions of the acetylene molecule with framework: d [O(-CO_2_)···H(C_2_H_2_)]=2.252 Å, d [H(-NH_2_)···(C_2_H_2_)]=2.856 Å). (**c**) Pore size distribution (PSD) of UTSA-100a. PSD was calculated using the well-known method by Gubbins *et al*.^58^. The van der Waals diameters of the framework atoms were adopted from the Cambridge Crystallographic Center. (**d**) Pore size variation along the pore channel (in *c* axis direction), within the crystal unit cell of UTSA-100a. Colour scheme: Cu, bright green; O, red; N, blue; C, orchid (green in acetylene); and H, yellow.

To correlate the excellent C_2_H_2_/C_2_H_4_ separation performance of UTSA-100a to its structure, we carried out more detailed pore structure analysis. As mentioned earlier, the overall MOF pore size is centred at 4.3 Å, with rather narrow distribution (Fig. 5c). Gas adsorption and diffusion would thus take place predominantly within individual channel pores, with interchannel diffusion limited by the narrow window opening between adjacent channels and the size of gas molecules. The pore size variation along the pore channel (in crystallography *c* axis direction) is shown in Fig. 5d. Clearly, the pore limiting size (for the access of guest gas molecule into the MOF crystal) is 3.96 Å, while the largest cavity within the channel is 4.6 Å in diameter. Note that the empirical kinetic diameters of C_2_H_2_/C_2_H_4_ are ∼3.3/4.2 Å (3.32 × 3.34 × 5.70 Å^3^ for C_2_H_2_ versus 3.28 × 4.18 × 4.84 Å^3^ for C_2_H_4_), respectively^46^,^47^,^48^,^49^. For ethylene, its kinetic diameter is slightly larger than the channel pore opening of UTSA-100a, and much larger than the interchannel window size. These two factors may significantly hinder/block the ethylene adsorption and diffusion in the MOF structure, leading to the much lower ethylene uptakes than acetylene ones.

To further understand the acetylene adsorption in UTSA-100a, we performed detailed computational investigations (Supplementary Methods). We first optimized the bare UTSA-100a structure by first-principles DFT-D (dispersion-corrected density-functional theory) calculations, where van der Waals interactions were corrected by empirical *r*^−6^ terms^50^. The optimized structure is fairly close to the experimentally determined structure. We then introduced acetylene molecules to various locations of the channel pore, and further optimized the ‘UTSA-100a+C_2_H_2_' structures using DFT-D. Interestingly, the guest acetylene molecules all get relaxed to a particular adsorption sites. In Fig. 5b, we plot this preferred acetylene adsorption location. The acetylene sits right at the small cage connecting two adjacent channel pores. The relatively strong binding clearly comes from multiple-point interactions of the molecule with framework (particularly, the metal center O, the linker tetrazole rings and –NH_2_ groups, Fig. 5b). These binding sites are typically classified as specific and/or strong sites for gas recognitions^29^. The interactions between the metal center O with C_2_H_2_ can be assigned as hydrogen bonding interactions. Given the fact that aromatic –NH_2_ has weak basicity, while C_2_H_2_ has weak acidity (pKa=25)^51^, there might exist weak acid–base interactions between –NH_2_ groups and C_2_H_2_ molecules. Because C_2_H_2_ is more acidic than C_2_H_4_ (pKa=44)^51^, the –NH_2_ groups have stronger interactions with C_2_H_2_ than C_2_H_4_, which enforces the selective binding of UTSA-100a for C_2_H_2_ than C_2_H_4_ as well. It is suggested that the relatively narrower pore size allows and reinforces one acetylene molecule to interact mutually with –NH_2_ group and one metal center O atom. We note that the adsorbed acetylene is slightly distorted, with an induced dipole moment. The H–C–C bond angle of acetylene is 178.8°, comparable to that of acetylene adsorbed on the open-Cu site in HKUST-1 (∼178°)^40^. The static acetylene binding energy, derived from the DFT-D calculation, is ∼31.3 kJ mol^−1^. This value (without considering the thermal correction, which is typically a few kJ mol^−1^) is somewhat larger than the experimental *Q*_st_ value but still reasonable, considering the accuracy limitation of the DFT-D approach.

## Discussion

Removal of acetylene from ethylene/acetylene mixtures containing 1% acetylene is a very important but challenging industrial separation task. It has been speculated that adsorption-based porous materials could be the good alternative for the highly efficient removal of acetylene from ethylene steam, but have not been fully fulfilled. To evaluate a porous material for acetylene removal from ethylene, adsorption selectivity and saturation uptake capacity have been deemed as two most important criteria, and high values for both of them are needed to achieve high effectiveness and high efficiency for acetylene removal. However, which factor plays the dominant role depends on the composition of gas mixtures. For the ethylene/acetylene mixtures containing 1% acetylene, adsorption selectivity will be enough more important than acetylene adsorption capacity. Comparing with other five well-known MOFs (M'MOF-3a, MgMOF-74, CoMOF-74, FeMOF-74 and NOTT-300), the significantly superior performance of UTSA-100a in removing acetylene from ethylene/acetylene mixtures containing 1% acetylene is attributable to the collaboration of high adsorption selectivity and high uptake capacity at ambient conditions. From the structure point of view, UTSA-100a has suitable pores and opening windows to enforce its high sieving effects and thus high adsorption selectivities, while the suitable cages and immobilized functional sites such –NH_2_ further maximize the acetylene uptakes. It is speculated that weak acid–base interactions between –NH_2_ and C_2_H_2_ molecules also play the important roles for the preferential binding of UTSA-100a with C_2_H_2_ over C_2_H_4_. Incorporation of stronger basic sites such as alkaneamines into porous MOFs might significantly differentiate their interactions with C_2_H_2_ over C_2_H_4_, leading to even more efficient MOF materials for C_2_H_2_/C_2_H_4_ separations in the future.

It is to be noted that our comparisons of different MOFs are for ethylene/acetylene mixtures containing 1% acetylene that is representative of compositions encountered in industry. If we were to compare the performance of UTSA-100a, NOTT-300 and FeMOF-74 for 50/50 ethylene/acetylene mixtures, the conclusions are different because in this case, capacity considerations would be very important. Supplementary Figure 11 presents a comparison of breakthroughs for UTSA-100a, NOTT-300, and FeMOF-74 for 50/50 ethylene/acetylene mixtures. In this case the performance of NOTT-300 and UTSA-100a are nearly the same. The best separations are achieved with FeMOF-74 that has the highest capacity to adsorb acetylene. These results also underscore the need for a proper evaluation of MOFs using transient breakthroughs. Comparisons based pure on selectivities may lead to wrong conclusions.

In conclusion, we have prepared a microporous MOF (UTSA-100) for highly efficient removal of acetylene from ethylene/acetylene mixtures containing 1% acetylene. Experimental and computational simulation results demonstrate the high efficiency of UTSA-100a in the removal of acetylene from ethylene/acetylene mixtures containing 1% acetylene, which is a very important but challenging industrial separation task. The results of this research have important significance on the practical design and preparation of porous materials for light hydrocarbon separations. It will also provide some guidance on the design and synthesis of microporous MOFs for other gas separations.

## Methods

### Materials and measurements

Commercially available reagents were purchased in high purity and used without further purification. 5-(5-amino-1*H*-tetrazol-1-yl)-1,3-benzenedicarboxylic acid (H_2_ATBDC) was synthesized according to the literature method^52^ (Supplementary Fig. 1). ^1^H NMR and ^13^C NMR spectra were obtained using a Varian INOVA 500 MHz spectrometer at room temperature. FTIR spectra were performed on a Bruker Vector 22 spectrometer at room temperature. Thermal gravimetric analysis (TGA) was performed under a nitrogen atmosphere with a heating rate of 3 °C min^−1^ using a Shimadzu TGA-50 thermogravimetric analyzer. PXRD patterns were measured by a Rigaku Ultima IV diffractometer operated at 40 kV and 44 mA with a scan rate of 1.0 deg min^−1^.

### Synthesis of 5-(1*H*-tetrazol-1-yl)-1,3-benzenedicarboxylic acid

Glacial acetic acid (10.0 ml) was added with stirring to a suspension of 5-aminoisophthalic acid (4.54 g, 0.025 mol), and sodium azide (1.79 g, 0.0275, mol) in triethyl orthoformate (14.2 ml, 0.075 mol), and the mixture was stirred at 80–90 °C for 6 h. The reaction mixture was cooled, and concentrated hydrochloric acid (4.2 ml, 0.05 mol) and water (12.5 ml) were added. The precipitated solid was separated by filtration, washed with water and dried. The obtained raw product was recrystallized from DMF. Yield: 82% (4.79 g). ^1^H NMR (500 MHz, *d*^6^-DMSO, p.p.m.): *δ* 13.78 (s, 2H, –CO_2_H), 10.33 (s, 1H, –N_4_CH), 8.65 (s, 2H, –C_6_H_3_), 8.55 (s, 1H, –C_6_H_3_).

### Synthesis of 5-(cyanoamino)-1,3-benzenedicarboxylic acid

DMSO (20.0 ml) was added dropwise with constant stirring to a suspension of 5-(1*H*-tetrazol-1-yl)-1,3-benzenedicarboxylic acid (4.68 g, 0.02 mol) in 22% aqueous KOH solution (12.0 ml). Gas evolution was observed, accompanied by self-heating of the reaction mixture. Stirring of the reaction mixture was continued for 2 days. The mixture was then diluted to 160 ml with water, acidified with concentrated hydrochloric acid to pH 3–4 and stored at 5–10 °C until precipitation of solid. The obtained product was filtered off and dried in vacuum. Another portion of product was reprecipitated from the filtrate through salting out method using sodium salt. Yield: 93% (3.83 g). ^1^H NMR (500 MHz, d^6^-DMSO, p.p.m.): *δ*=7.67 (s, 1H, –C_6_H_3_), 7.40 (s, 2H, –C_6_H_3_).

### Synthesis of 5-(5-amino-1*H*-tetrazol-1-yl)-1,3-benzenedicarboxylic acid

A suspension of 5-(cyanoamino)-1,3-benzenedicarboxylic acid (2.06 g, 0.01 mol), sodium azide (0.98 g, 0.015 mol) and ammonium chloride (1.07 g, 0.02 mol) in DMF (25 ml) was stirred at 70–80 °C for 6 h, after which water (100 ml) was added to the reaction mixture. The white solid was precipitated through salting out method using sodium salt. The obtained product was filtered off, and dried in vacuum. Yield: 82% (2.04 g). ^1^H NMR (500 MHz, D_2_O, p.p.m.): *δ*=8.47 (s, 1H, –C_6_H_3_), 8.14 (s, 2H, –C_6_H_3_) (Supplementary Fig. 2). ^13^C NMR (D_2_O, p.p.m.): *δ*=173.00, 138.49, 132.35, 130.43, 126.94 (Supplementary Fig. 3).

### Synthesis of UTSA-100

A mixture of CuCl_2_·2H_2_O (34 mg, 0.2 mmol) and the organic linker H_2_ATBDC (50 mg, 0.2 mmol) was dispersed into an 8 ml mixed solvent (DMF/MeOH, 5/3, v/v) in a screw-capped vial (20 ml). And five drops of HBF_4_ (48% w/w aqueous solution) were added. The suspension was sonicated until homogenous. The vial was capped and heated in an oven at 80 °C for 24 h. Green block crystals were obtained by filtration and washed with DMF several times to afford UTSA-100. IR (neat, cm^−1^): 1,739w; 1,630s; 1,596m; 1,490w; 1,457m; 1,377s; 1,255m; 1,128w; 1,097m; 1,062w; 908m; 780m; 725s; 677m; 661m.

### Gas sorption studies

A Micromeritics ASAP 2020 surface area analyzer was used to measure gas adsorption isotherms. To remove all the guest solvents in the framework, the fresh sample of UTSA-100 was guest exchanged with dry acetone at least 10 times, filtered and degassed at room temperature (296 K) for one day, and then at 358 K for another 3 days until the outgas rate was 5 μmHg min^−1^ before measurements. A sample of activated UTSA-100a (100–150 mg) was used for the sorption measurement and was maintained at 77 K with liquid nitrogen, at 273 K with an ice-water bath. As the center-controlled air conditioner was set up at 23 °C, a water bath was used for adsorption isotherms at 296 K.

### Fitting of pure component isotherms

Experimental data on pure component isotherms for acetylene and ethylene in UTSA-100a were measured at temperatures of 273 and 296 K. The pure component isotherm data for acetylene and ethylene were fitted with the dual-Langmuir–Freundlich isotherm model


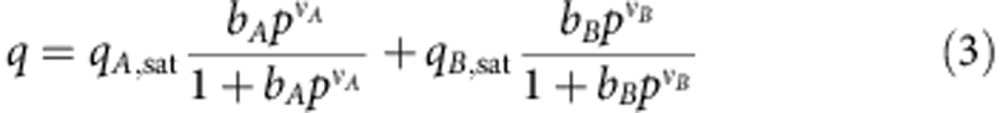


with *T*-dependent parameters *b*_A_ and *b*_B_


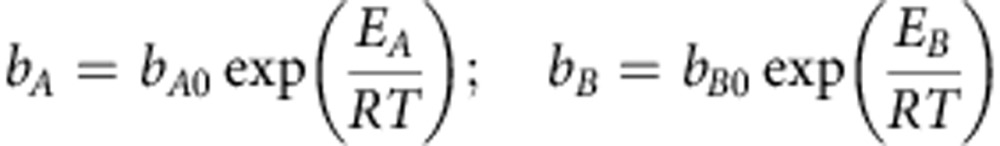


The fitted parameter values are presented in Supplementary Table 2.

For FeMOF-74, the dual-site Langmuir–Freundlich parameters are from Bloch *et al*.^38^; for convenience, the parameters are summarized in Supplementary Table 3. For NOTT-300, the isotherm data at 293 K were fitted with a single-site Langmuir isotherm model; the fit parameters are specified in Supplementary Table 4. Supplementary Figure 9 presents a comparison of component loadings for acetylene and ethylene at 293 K in NOTT-300 with 1-site Langmuir isotherm fits. The Langmuir fits are of good accuracy. For all other MOFs, the isotherm data are from He *et al*.^39^.

### Transient breakthrough of ethylene/acetylene mixtures in fixed-bed adsorbers

The performance of industrial fixed-bed adsorbers is dictated by a combination of adsorption selectivity and uptake capacity. For a proper comparison of various MOFs, we perform transient breakthrough simulations using the simulation methodology described in the literature^53^,^54^,^55^. For the breakthrough simulations, the following parameter values were used: framework density, *ρ* (1,146 kg m^−3^); length of packed bed, *L* (0.12 m); voidage of packed bed, *ɛ* (0.75); superficial gas velocity at inlet, *u* (0.00225, m s^−1^). For breakthrough simulations with NOTT-300, we use calculated the framework density from the crystal structure information provided in the paper of Yang *et al*.^42^; the resultant value is *ρ* (1,062 kg m^−3^). The framework densities for all other MOFs are available in the papers by Bloch *et al*.^38^ and He *et al*.^39^. The transient breakthrough simulation results are presented in terms of a dimensionless time, *τ*, defined by dividing the actual time, *t*, by the characteristic time, 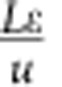.

### Column breakthrough tests

The breakthrough separation experiments were conducted on a home-made apparatus (Supplementary Fig. 12) with a set-up similar to what was described in Yaghi's paper^56^. In a typical experiment, 1.00 g of UTSA-100a powders were thoroughly ground and packed into a quartz column (5.8 mm inner diameter × 150 mm) with quartz wool filling the void space. The sample was *in situ* activated under vacuum (6.5 × 10^−4^ Pa) at 353 K to remove adsorbed molecules and make the active sites accessible. The sample was then purged with He flow (2.0 ml min^−1^) for 1 h while the temperature of the column was decreased to 296 K. The mix gas (C_2_H_2_:C_2_H_4_=1: 99 by volume) flow was then introduced at 2.0 ml min^−1^. Effluent from the column was monitored using a mass spectrometer.

Caution: Because of its wide flammability limits and a potential for explosive decomposition^57^, acetylene should be handled with care.

## Additional information

**Accession codes:** The X-ray crystallographic coordinates for structures reported in this Article have been deposited at the Cambridge Crystallographic Data Centre (CCDC), under deposition number CCDC 1044083. These data can be obtained free of charge from The Cambridge Crystallographic Data Centre via www.ccdc.cam.ac.uk/data_request/cif.

**How to cite this article:** Hu, T.-L. *et al*. Microporous metal–organic framework with dual functionalities for highly efficient removal of acetylene from ethylene/acetylene mixtures. *Nat. Commun.* 6:7328 doi: 10.1038/ncomms8328 (2015).

## Supplementary Material

Supplementary Figures, Supplementary Tables, Supplementary Methods and Supplementary References Supplementary Figures 1-13, Supplementary Tables 1-5, Supplementary Methods and Supplementary References

Supplementary Data 1 Crystal data.

## Figures and Tables

**Table 1:** Adsorption data.

	**M'MOF-3a**	**MgMOF-74**	**CoMOF-74**	**FeMOF-74**	**NOTT-300**	**UTSA-100a**
Surface area (m^2^ g^−1^; BET)	110	927	1,018	1,350	1,370	970
Pore volume (cm^3^ g^−1^)	0.165	0.607	0.515	0.626	0.433	0.399
Framework density(kg m^−3^)	1,023	909	1,169	1,126	1,062	1,146
Size of pore window (Å)	3.4 × 4.8	11 × 11	11 × 11	11 × 11	6.5 × 6.5	4.3 × 4.3
C_2_H_2_ uptake at 1.0 bar (mmol g^−1^)	1.90	8.37	8.17	6.80*	6.34†	4.27
C_2_H_4_ uptake at 1.0 bar (mmol g^−1^)	0.40	7.45	7.02	6.10*	4.28†	1.66
C_2_H_2_/C_2_H_4_ uptake ratio	4.75	1.12	1.16	1.11	1.48	2.57
Selectivity for C_2_H_2_/C_2_H_4_‡	24.03	2.18	1.70	2.08	2.17	10.72
*Q*_st_ (C_2_H_2_, kJ mol^−1^)§	25	41	45	46	32	22

BET, Brunauer, Emmett and Teller.

Summary of the adsorption data for acetylene and ethylene in representative MOFs at 296 K^38^,^39^,^42^.

^*^At temperature of 318 K.

^†^At temperature of 293 K.

^‡^IAST analysis for ethylene/acetylene mixtures containing 1% acetylene at 100 kPa.

^§^Q_st_ values at low surface coverage.
