# Supplementary material for: Microporous metal–organic framework with dual functionalities for highly efficient removal of acetylene from ethylene/acetylene mixtures
Source: Nat Commun. 2015 Jun 4;6:7328. doi: 10.1038/ncomms8328 (PMC4468854; doi:10.1038/ncomms8328)
Supplement: Supplementary Figures, Supplementary Tables, Supplementary Methods and Supplementary References — Supplementary Figures 1-13, Supplementary Tables 1-5, Supplementary Methods and Supplementary References [file ncomms8328-s1.pdf]

Supplementary Information

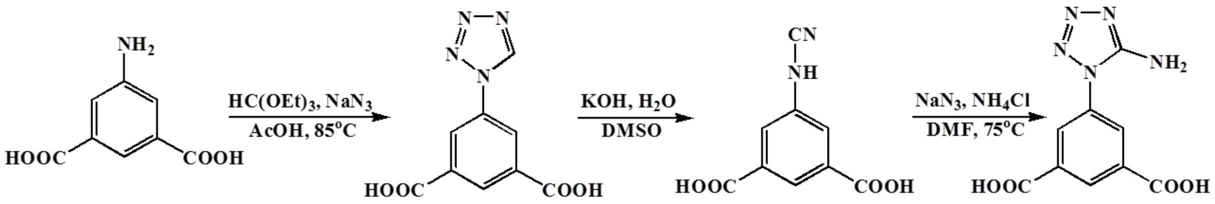

Supplementary Figure 1 │ Synthetic routes to the organic linker H_2ATBDC.

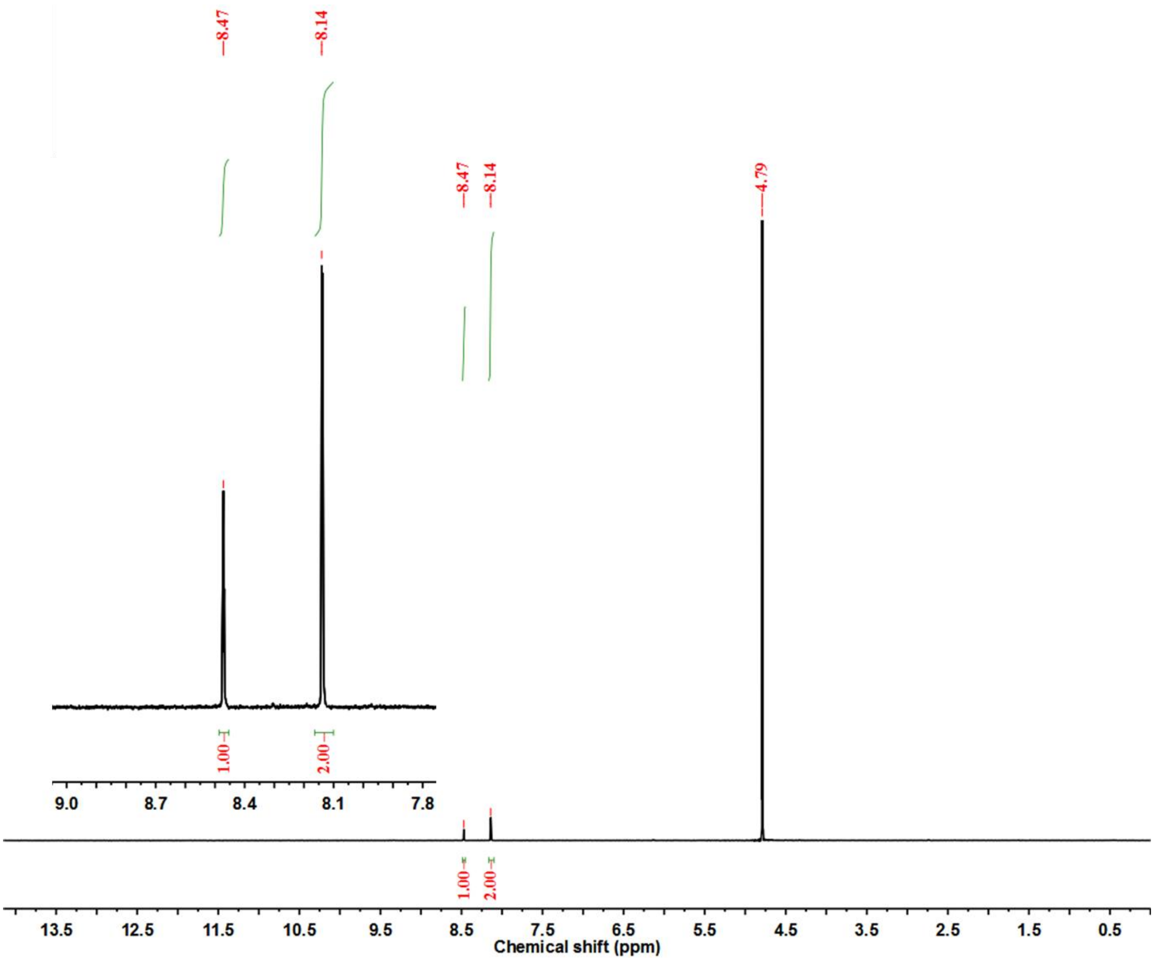

Supplementary Figure 2 │ ^{1}H NMR (D_2O, 500MHz) spectrum of H_2ATBDC.

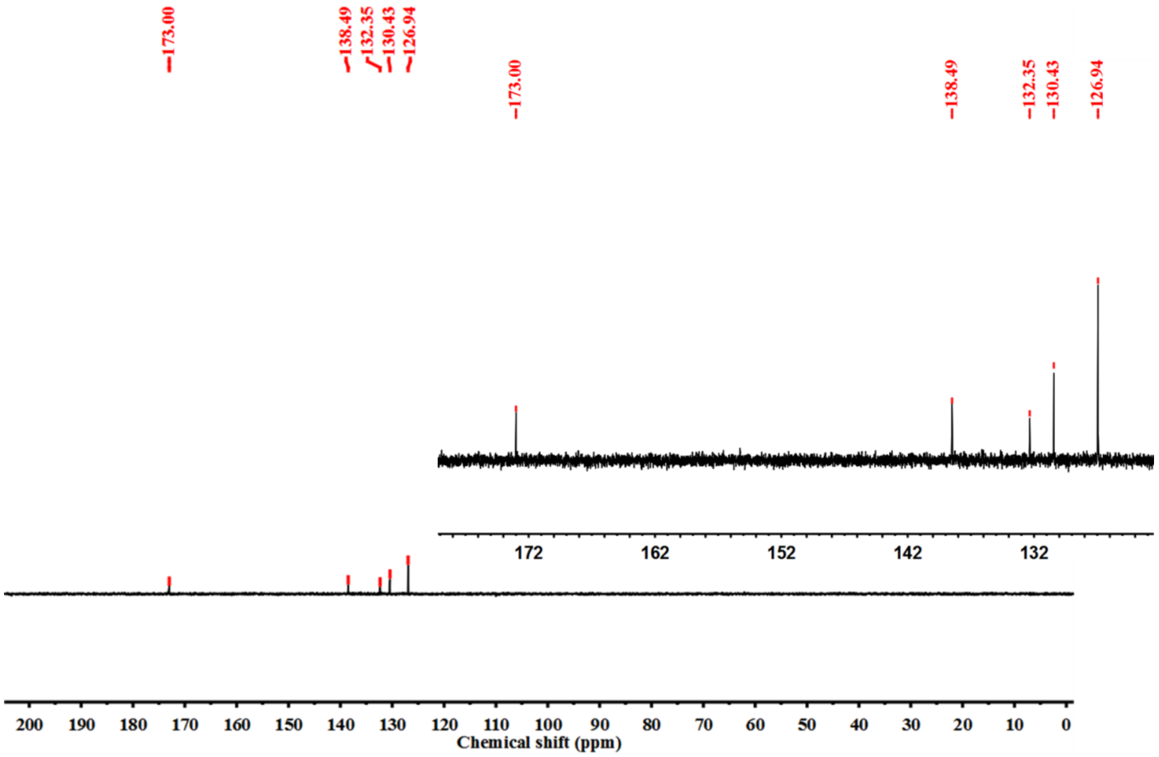

Supplementary Figure 3 | ^{13}C NMR (D_2O, 500MHz) spectrum of H_2ATBDC.

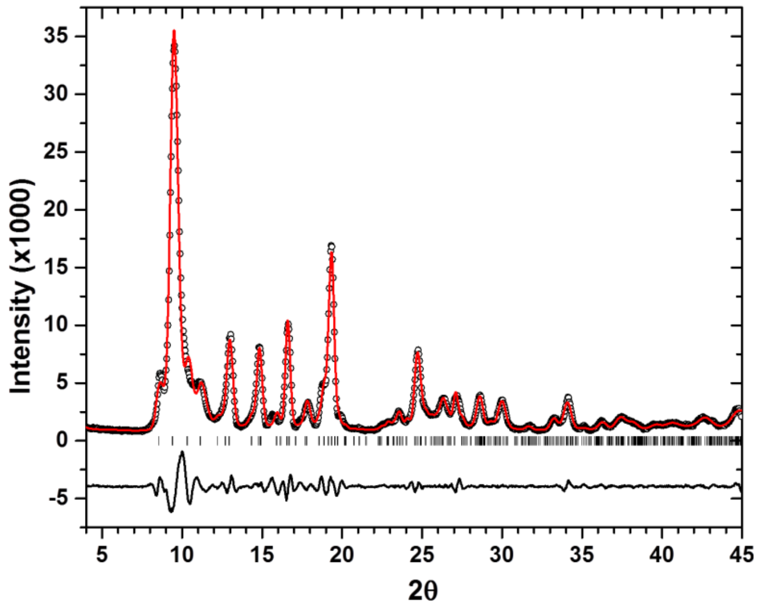

Supplementary Figure 4 │Powder X-ray diffraction (PXRD) patterns of UTSA-100. Experimental (circles), Le Bail fitted (line), and difference (line below observed and calculated patterns) PXRD profile for as-synthesized UTSA-100 at 298 K (Cu Ka radiation). Vertical bars indicate the calculated positions of Bragg peaks. Refined lattice parameters: a=12.369(3) Å, b=14.509(3) Å and c=20.755(6) Å. Goodness of fit: R_p=0.0677, R_{wp}=0.0871.

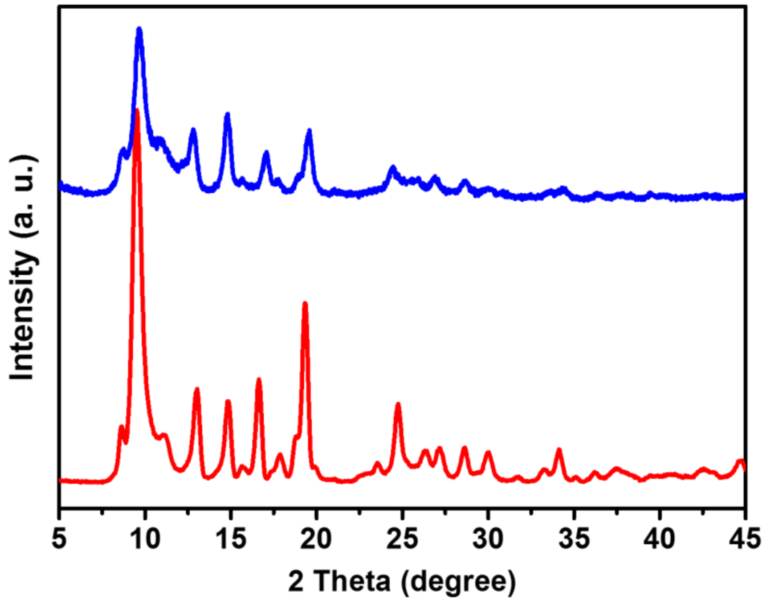

Supplementary Figure 5 │ Powder X-ray diffraction (PXRD) patterns of as-synthesized UTSA-100 (red) and activated UTSA-100a (blue).

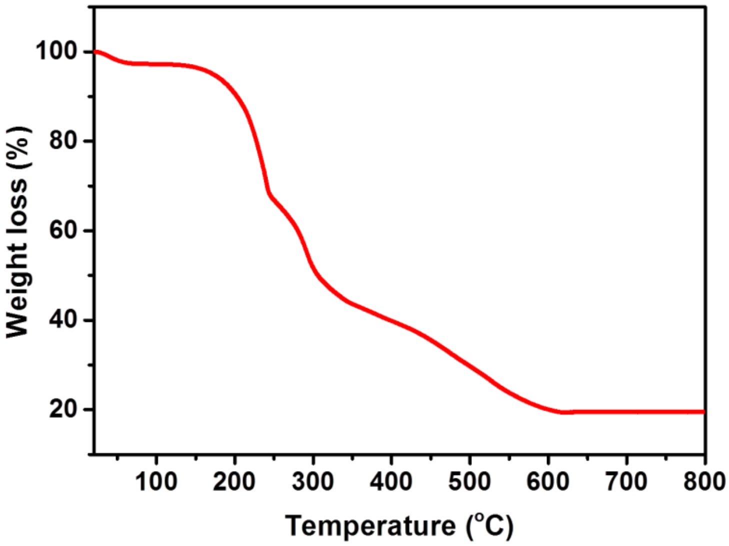

Supplementary Figure 6 │ TGA curve of UTSA-100.

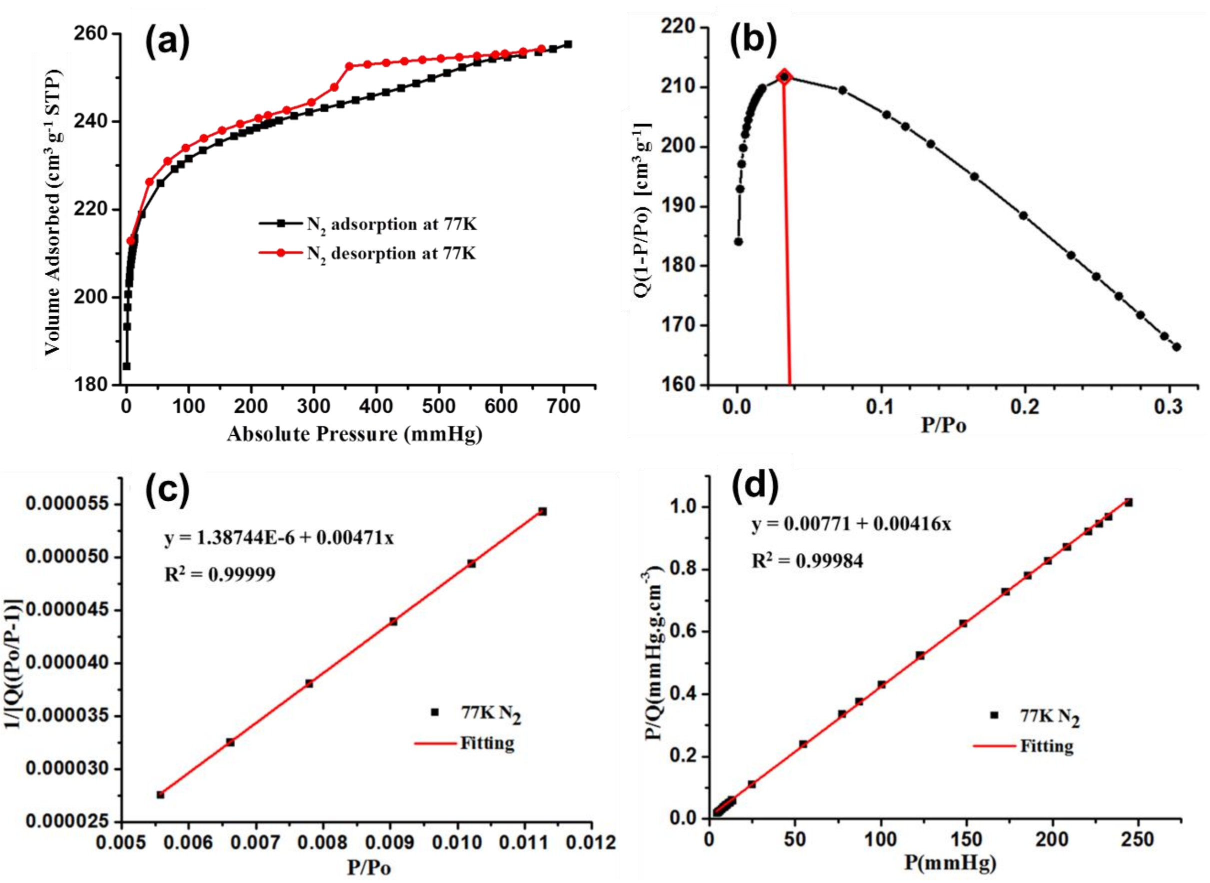

S_{BET} = [1/(0.00471-0.00000138744)]/22414 × 6.023 × 10^{23} × 0.170 × 10^{-18} = 970 m^2g^{-1}.

$S_{Langmuir}$ = [(1/0.00416)/22414] × 6.023 × 10^{23} × 0.170 × 10^{-18} = 1098 m^2g^{-1}.

Supplementary Figure 7 │N_2 sorption isotherm and the surface areas. (**a**) N_2 sorption isotherm of UTSA-100a at 77K. (**b**) Plot of the term Q(1-P/P_0) *vs* P/P_0. (**c**) The BET and Langmuir (**d**) surface areas of UTSA-100a obtained from the N_2 adsorption isotherm at 77 K.

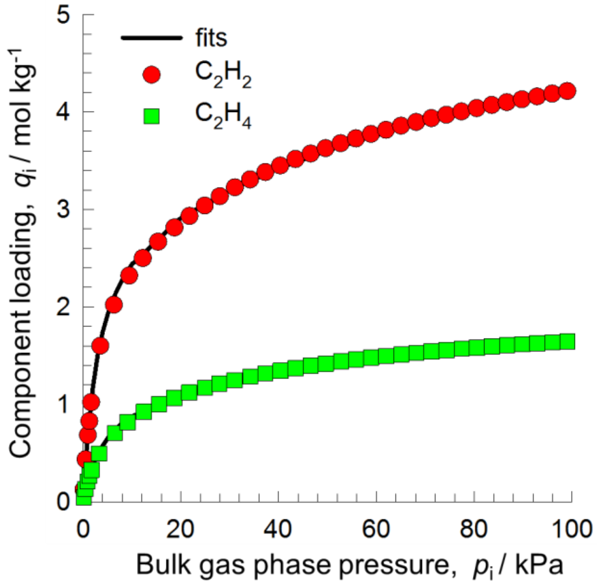

Supplementary Figure 8 │ Comparison of component loadings for C_2H_2 and C_2H_4 at 296 K in UTSA-100a with dual-Langmuir-Freundlich isotherm fits.

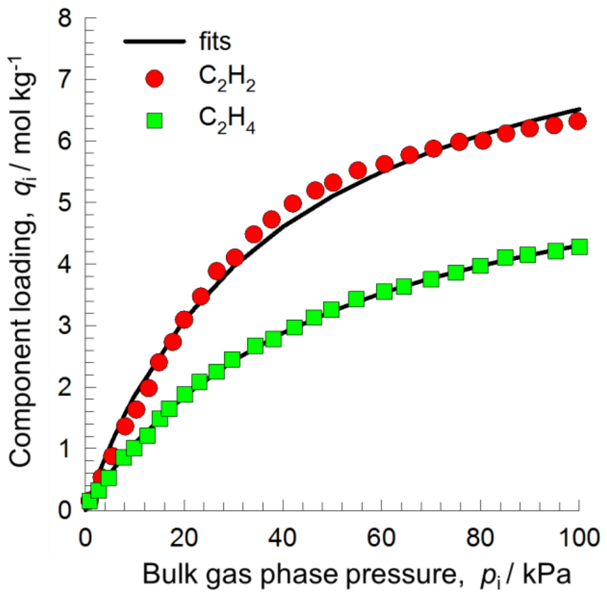

Supplementary Figure 9 │ Comparison of component loadings for C_2H_2 and C_2H_4 at 293 K in NOTT-300 with 1-site Langmuir isotherm fits.

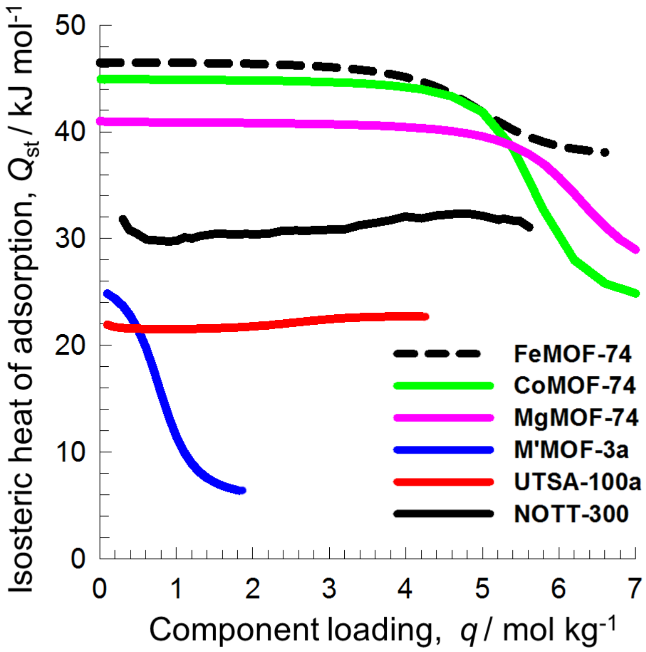

Supplementary Figure 10 │ Comparison of the heats of adsorption of C_2H_2 in various MOFs. The Q_{st} data for NOTT-300 has been scanned from Yang et al.[1] for discrete points; this explains the non-smooth nature of the curve. The data for other MOFs has been taken from He et al.[2]

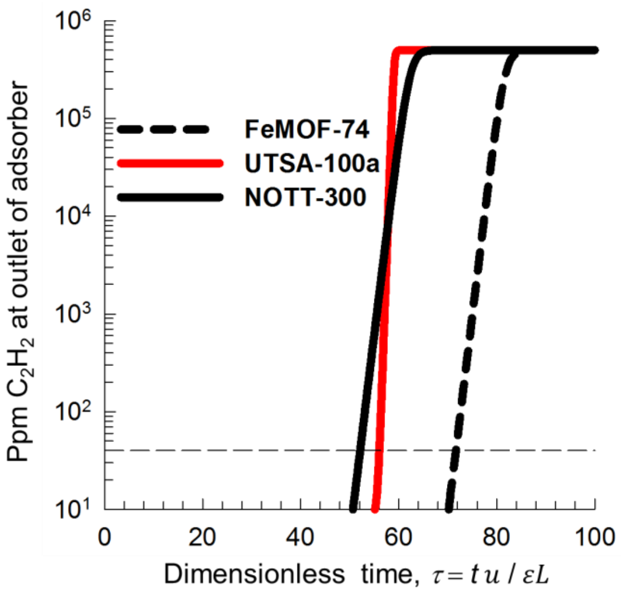

Supplementary Figure 11 │ Comparison of breakthroughs for three representative MOFs for 50:50 acetylene/ethylene mixtures at 296 K and 100 kPa.

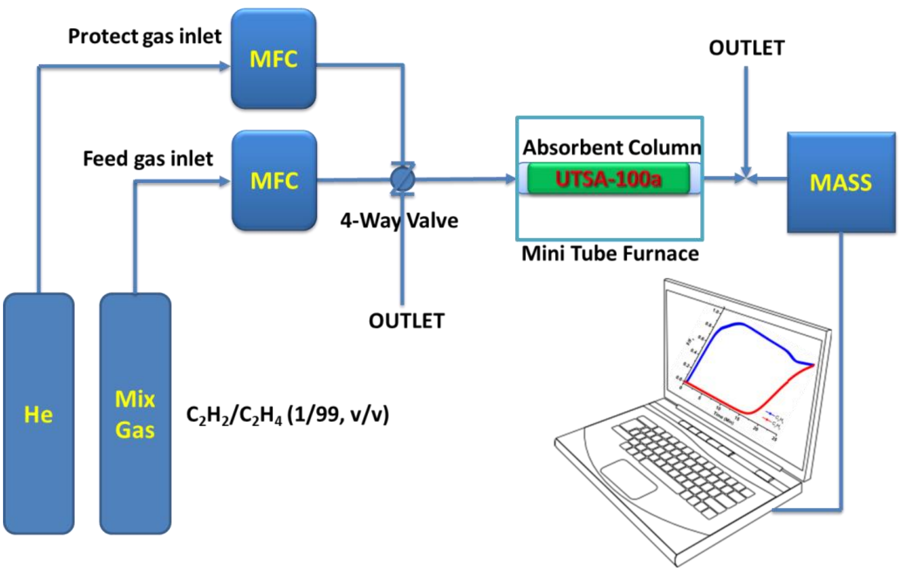

Supplementary Figure 12 │ Schematic illustration of the apparatus used for the breakthrough experiments.

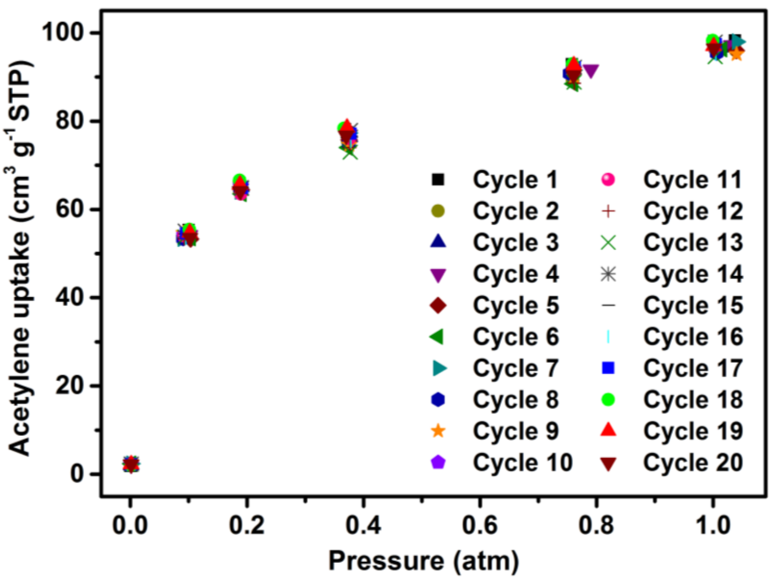

Supplementary Figure 13 │ Twenty cycles of acetylene uptakes for UTSA-100a at 296 K.

Supplementary Table 1 │ Crystallographic data and structure refinement results for UTSA-100 (from single-crystal X-ray diffraction analysis on the as-synthesized sample).

	**UTSA-100**
Formula	$C_9H_5CuN_5O_4$
Formula weight	310.72
Temperature/K	100.33(10)
Crystal system	Orthorhombic
Space group	*Pbcn*
a(Å)	12.1905(11)
b(Å)	14.4177(13)
c(Å)	20.4894(19)
α(°)	90.00
β(°)	90.00
γ(°)	90.00
V(Å$_3$)	3601.2(6)
Z	8
D_{calcd}(g cm^{-3})	1.146
μ(mm^{-1})	1.858
F(000)	1240
Crystal size/mm^3	0.15× 0.10× 0.08
GOF	0.961
R_{int}	0.1589
R_1,wR_2[I>=2σ (I)]	0.0871, 0.2303
R_1, wR_2[all data]	0.1106, 0.2569
Largest diff. peak and hole (e Å^{-3})	2.187, -0.822

Supplementary Table 2 │ Dual-Langmuir-Freundlich parameter fits for UTSA-100a.

	Site A				Site B			
	$q_{A,sat}$ mol kg^{-1}	b_{A0} $Pa^{-\nu_i}$	E_A kJ mol^{-1}	ν_A dimensionless	$q_{B,sat}$ mol kg^{-1}	b_{B0} $Pa^{-\nu_i}$	E_B kJ mol^{-1}	ν_B dimensionless
**C_2H_2**	2	1.19×10^{-9}	26.5	1.25	15	2.42×10^{-6}	12.3	0.54
**C_2H_4**	1.8	7.19×10^{-12}	33	1	1.15	3.37×10^{-10}	33	1

Note: q_i component molar loading of species i, mol kg^{-1}; b Langmuir-Freundlich constant, $Pa^{-\nu_i}$

ν exponent in dual-Langmuir-Freundlich isotherm, dimensionless; q_{sat} saturation loading, mol kg^{-1}

Supplementary Table 3 │ Dual-Langmuir-Freundlich parameter fits for FeMOF-74.

	Site A			Site B		
	$q_{i,A,sat}$ mol kg^{-1}	$b_{i,A}$ $Pa^{-\nu_i}$	$\nu_{i,A}$ dimensionless	$q_{i,B,sat}$ mol kg^{-1}	$b_{i,B}$ $Pa^{-\nu_i}$	ν_B dimensionless
**C_2H_2**	5.3	1.086×10^{-3}	1	3.6	8.69×10^{-6}	1
**C_2H_4**	3.6	3.71×10^{-4}	1.1	3.3	8.29×10^{-5}	1

The fit parameters are for 318 K from the paper by Bloch et al.[3]

Supplementary Table 4 │ Single-site Langmuir fits for NOTT-300 at 293 K.

	$q_{A,sat}$ mol kg^{-1}	b_{A0} Pa^{-1}
**C_2H_2**	9	2.62×10^{-5}
**C_2H_4**	6.4	2.06×10^{-5}

The isotherm data from Yang et al.[1] at 293 K are fitted.

Supplementary Table 5 │ Breakthrough calculations for separation of C_2H_2/C_2H_4 mixture containing 1 mol% C_2H_2 at 296 K.

	**Dimensionless Breakthrough Time, τ_{break}**	**Adsorbed Amount of C_2H_2 during 0 - τ_{break}, mmol L^{-1}**
**UTSA-100a**	113.7	137.6
**MgMOF-74**	84.0	101.3
**FeMOF-74**	89.0	100.7
**CoMOF-74**	77.4	93.3
**M'MOF3a**	60.2	72.3
**NOTT-300**	56.3	68.3

The data for FeMOF-74 is at a temperature of 318 K; this is the lowest temperature used in the isotherm measurements of Bloch et al.[3] The data for NOTT-300 is at 293 K, for which the isotherm data is available in Yang et al.[1] The product gas stream contains less than 40 ppm C_2H_2.

Supplementary Methods:

Single-crystal X-ray crystallography. The crystal data were collected on an Agilent Supernova CCD diffractometer equipped with agraphite-monochromatic enhanced Cu Kα radiation (λ = 1.54184Å) at 100K. The datasets were corrected by empirical absorption correction using spherical harmonics, implemented in the SCALE3 ABSPACK scaling algorithm. The structure was solved by direct methods and refined by full matrix least-squares methods with the SHELX-97 program package[4]. The solvent molecules in the compound are highly disordered. The SQUEEZE subroutine of the PLATON software suit was used to remove the scattering from the highly disordered guest molecules[5,6]. The resulting new files were used to further refine the structures. The H atoms on C and N atoms were generated geometrically.

First-principles DFT-D calculations. First-principles calculations based on density-functional theory were performed using the PWSCF package[7]. A semi-empirical addition of dispersive forces to conventional DFT[8] was included in the calculation to account for van der Waals interactions. We used Vanderbilt-type ultrasoft pseudopotentials and the generalized gradient approximation (GGA) with the Perdew-Burke-Ernzerhof (PBE) exchange correlation. A cutoff energy of 544 eV and a 2x2x2 *k* sampling were sufficient for the total energy to converge within 0.5 meV/atom. We first optimized the bare UTSA-100a structure. C_2H_2 molecules were then introduced to various locations in the channel pore of the optimized UTSA-100a structure, followed by full structural relaxations. To obtain the gas binding energies, a free C_2H_2 molecule placed in a supercell with the same cell dimensions was also relaxed as a reference. The static binding energy was calculated using: $E_B = [E(MOF) + nE(C_2H_2) - E(MOF + nC_2H_2)]/n$.

Disclaimer: Certain commercial equipment, instruments, or materials are identified in this paper to foster understanding. Such identification does not imply recommendation or endorsement by the National Institute of Standards and Technology, nor does it imply that the materials or equipment identified are necessarily the best available for the purpose.

Supplementary References

1. Yang, S. *et al*. Supramolecular binding and separation of hydrocarbons within a functionalized porous metal–organic framework. *Nat. Chem.* **7**, 121–129 (2015).
2. He, Y., Krishna, R. & Chen, B. Metal–organic frameworks with potential for energy–efficient adsorptive separation of light hydrocarbons. *Energy Environ. Sci.* **5**, 9107-9120 (2012).
3. Bloch, E. D. *et al*. Hydrocarbon separations in a metal–organic framework with open iron(II) coordination sites. *Science* **335**, 1606–1610 (2012).
4. Sheldrick, G. M. Program for Structure Refinement. Germany, (1997).
5. Spek, A. L. PLATON: The University of Utrecht: Utrecht, The Netherlands (1999).
6. Spek, A. L. Single-crystal structure validation with the program PLATON. *J. Appl. Crystallogr.* **36**, 7–13 (2003).
7. Giannozzi, P. *et al*. QUANTUM ESPRESSO: A modular and open-source software project for quantum simulations of materials. *J. Phys.: Condens. Matter* **21**, 395502 (2009).
8. Barone, V. *et al*. Role and effective treatment of dispersive forces in materials: polyethylene and graphite crystals as test cases. *Comput. Chem.* **30**, 934–939 (2009).
